# Supplementary material for: The interactome of intact mitochondria by cross-linking mass spectrometry provides evidence for coexisting respiratory supercomplexes
Source: Mol Cell Proteomics. 2017 Dec 8;17(2):216–32. doi: 10.1074/mcp.RA117.000470 (PMC5795388; doi:10.1074/mcp.RA117.000470)
Supplement: Supplemental Data [file supp_RA117.000470_133922_0_supp_23978_fzffwf.zip › spectra_annotation/mito_spectra_annotation/378-1-2-1-15-1.pdf]

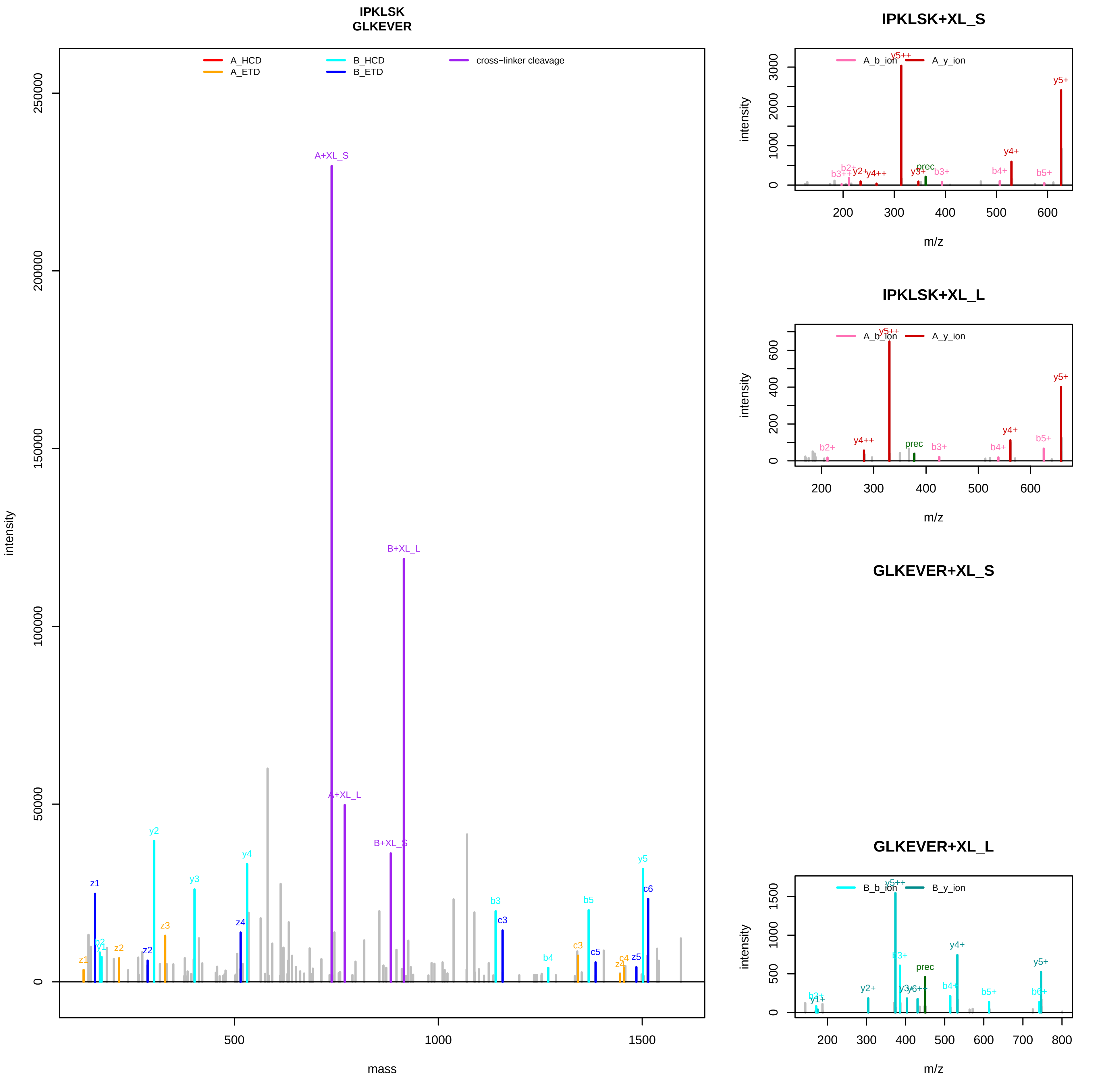
IPKLSK
GLKEVER
A_HCD
A_ETD
B_HCD
B_ETD
cross−linker cleavage
A+XL_S
B+XL_L
A+XL_L
B+XL_S
z1 z2 z3 z4 c3 c4
z1 z2 z4 z5 c3 c5 c6
y1 y2 y3 y4 y5 b2 b3 b4 b5
intensity
mass
0
50000
100000
150000
200000
250000
500
1000
1500
IPKLSK+XL_S
A_b_ion
A_y_ion
y5++ y5+ y4+ y4++ y3+ y2+ b2+ b3++ b3+ b4+ b5+ prec
0
1000
2000
3000
200
300
400
500
600
m/z
IPKLSK+XL_L
A_b_ion
A_y_ion
y5++ y5+ y4+ y4++ b2+ b3+ b4+ b5+ prec
0
200
400
600
200
300
400
500
600
m/z
GLKEVER+XL_S
GLKEVER+XL_L
B_b_ion
B_y_ion
y5++ y5+ y4+ y3+ y6++ y2+ y1+ b2+ b3+ b4+ b5+ b6+ prec
0
500
1000
1500
200
300
400
500
600
700
800
m/z
